# Association of Inadvertent 9-Valent Human Papillomavirus Vaccine in Pregnancy With Spontaneous Abortion and Adverse Birth Outcomes

**DOI:** 10.1001/jamanetworkopen.2021.4340

**Published:** 2021-04-05

**Authors:** Elyse O. Kharbanda, Gabriela Vazquez-Benitez, Malini B. DeSilva, Allison L. Naleway, Nicola P. Klein, Rulin C. Hechter, Jason M. Glanz, James G. Donahue, Lisa A. Jackson, Sangini S. Sheth, Victoria Greenberg, Lakshmi Panagiotakopoulos, Adamma Mba-Jonas, Heather S. Lipkind

**Affiliations:** 1 Department of Research, HealthPartners Institute, Minneapolis, Minnesota; 2 The Center for Health Research, Kaiser Permanente Northwest, Portland, Oregon; 3 The Vaccine Study Center, Kaiser Permanente Northern California, Oakland, California; 4 Department of Research and Evaluation, Kaiser Permanente Southern California, Pasadena, California; 5 Institute for Health Research, Kaiser Permanente Colorado, Denver, Colorado; 6 Marshfield Clinic Research Institute, Marshfield, Wisconsin; 7 Kaiser Permanente Washington, Health Research Institute, Seattle, Washington; 8 Department of Obstetrics, Gynecology, and Reproductive Sciences, Yale University School of Medicine, New Haven, Connecticut; 9 Centers for Disease Control and Prevention, Atlanta, Georgia; 10 Office of Biostatistics and Epidemiology, Center for Biologics Evaluation and Research, Division of Epidemiology, US Food and Drug Administration, Silver Spring, Maryland

## Abstract

**Importance:**

The 9-valent human papillomavirus (9vHPV) vaccine is recommended for individuals through age 26 years and may be administered to women up to age 45 years. Data on 9vHPV vaccine exposures during pregnancy are limited.

**Objective:**

To evaluate the associations between 9vHPV vaccine exposures during pregnancy or peripregnancy and selected pregnancy and birth outcomes (spontaneous abortion [SAB], preterm birth, small-for-gestational age [SGA] birth, and major structural birth defect).

**Design, Setting, and Participants:**

This cohort study analyzed data from 7 participating health systems in the Vaccine Safety Datalink. The cohort comprised pregnancies among girls and women aged 12 to 28 years that ended between October 26, 2015, and November 15, 2018. Singleton pregnancies that ended in a live birth, stillbirth, or SAB were included.

**Exposures:**

Vaccine exposure windows were distal (9vHPV or 4vHPV vaccine administered from 22 to 16 weeks before last menstrual period [LMP]), peripregnancy (9vHPV vaccine administered from 42 days before LMP until LMP), and during pregnancy (9vHPV vaccine administered from LMP to 19 completed weeks’ gestation). Primary comparisons were (1) girls and women with 9vHPV vaccine exposures during pregnancy vs those with 4vHPV or 9vHPV distal vaccine exposures, (2) girls and women with vaccine exposures peripregnancy vs those with 4vHPV or 9vHPV distal vaccine exposures, and (3) girls and women with 9vHPV vaccine exposures during pregnancy or peripregnancy vs those with 4vHPV or 9vHPV distal vaccine exposure.

**Main Outcomes and Measures:**

Spontaneous abortions were confirmed based on medical record review and adjudication. Preterm and SGA births were identified from electronic health record and birth data. Major structural birth defects were based on diagnostic codes using a validated algorithm. Inverse probability weighting was used to balance the covariates. Time-dependent covariate Cox proportional hazards regression models and Poisson regression were used to estimate the associations between 9vHPV vaccine exposures and pregnancy and birth outcomes.

**Results:**

The final cohort included 1493 pregnancies among girls and women with a mean (SD) maternal age of 23.9 (2.9) years. Of these pregnancies, 445 (29.8%) had exposures to the 9vHPV vaccine during pregnancy, 496 (33.2%) had exposures to the 9vHPV vaccine peripregnancy, and 552 (37.0%) had 4vHPV or 9vHPV distal vaccine exposures. The 9vHPV vaccine administered during pregnancy was not associated with increased risk for SAB (hazard ratio, 1.12; 95% CI, 0.66-1.93) compared with distal vaccine exposures. Findings were similar for 9vHPV vaccine exposures peripregnancy (relative risk [RR], 0.72; 95% CI, 0.42-1.24). Among live births (n = 1409), 9vHPV vaccine exposures during pregnancy were not associated with increased risks for preterm birth (RR, 0.73; 95% CI, 0.44-1.20) or SGA birth (RR, 1.31; 95% CI, 0.78-2.20). Results were similar regarding the association between 9vHPV vaccine exposures peripregnancy and preterm birth (RR, 0.72; 95% CI, 0.45-1.17) and SGA birth (RR, 1.10; 95% CI, 0.65-1.88). Birth defects were rare in all exposure groups, occurring in about 1% of live births with available infant data.

**Conclusions and Relevance:**

This study found that 9vHPV vaccine exposures during or around the time of pregnancy were uncommon and not associated with SABs or selected adverse birth outcomes. These findings can inform counseling for inadvertent 9vHPV vaccine exposures.

## Key Points

**Question:**

Is exposure to the 9-valent human papillomavirus (9vHPV) vaccine in pregnancy associated with adverse pregnancy or birth outcomes?

**Findings:**

In this cohort study of 1493 pregnancies, in adjusted analyses, during-pregnancy or peripregnancy exposures to 9vHPV vaccine were not associated with spontaneous abortion. Among live births, 9vHPV vaccine exposures during or around the time of pregnancy were not associated with adverse birth outcomes.

**Meaning:**

Results of this study suggest that, in an insured population, adverse events after exposure to 9vHPV vaccine during or around the time of pregnancy were uncommon; these findings can inform counseling after inadvertent 9vHPV vaccine exposures.

## Teaser

This cohort study examines the risks for spontaneous abortion, preterm birth, small-for-gestational age birth, and major structural birth defects among female teenagers and adults who received the human papillomavirus vaccine.

## Introduction

The quadrivalent human papillomavirus (4vHPV) vaccine was introduced in the US in 2006.^1^ The 9-valent human papillomavirus (9vHPV) vaccine was licensed in 2014 and was recommended in 2015 for routine vaccination at age 11 or 12 years, with catch-up vaccination through age 26 years.^2^ With expanded protection against oncogenic human papillomavirus (HPV) types (16, 18, 31, 33, 45, 52, and 58), the 9vHPV vaccine replaced the 4vHPV vaccine.^3^ Both the 4vHPV and 9vHPV vaccines have been found to prevent type-specific HPV infections and precancerous lesions.^4,5,6,7^ After licensure, the 4vHPV vaccine has been associated with decreased prevalence of HPV infections and their sequelae.^8,9,10,11^

In 2019, only 59% of girls in the US completed the HPV vaccine series by age 15 years.^12^ Depending on age at first dose, up to 3 doses of 9vHPV vaccine may be indicated by age 26 years. The 9vHPV vaccine is now licensed and may be considered for women 27 to 45 years of age.^13^ The Advisory Committee on Immunization Practices recommends that, in a known pregnancy, HPV vaccination should be administered after pregnancy; yet, pregnancy testing is not recommended before routine HPV vaccination in female individuals of reproductive age.^13^ Among female adolescents and young adults aged 13 to 27 years who received care in 7 large health systems from 2007 to 2013, exposures to the 4vHPV vaccine occurred during or around the time of 1.5% of pregnancies.^14^

Data from clinical trials and observational studies have not identified specific risks associated with 4vHPV vaccine exposures in pregnancy.^14,15,16,17,18,19^ Nevertheless, the Advisory Committee on Immunization Practices considers pregnancy a precaution for the 9vHPV vaccine largely on the basis of theoretical risks.^2^ In prelicensure clinical trials, Moreira et al^20^ found that among 172 women vaccinated within 30 days of the estimated date of conception, there was an increased rate of spontaneous abortion (SAB) after 9vHPV vaccine exposures around the time of pregnancy (20%) compared with 4vHPV vaccine exposures (9.2%). The small numbers were insufficient to fully evaluate the risks for adverse birth outcomes, and the potential increased risk for SAB after 9vHPV vaccine exposures requires additional investigation.

Since 2017, the 9vHPV vaccine has been the only HPV vaccine distributed in the US^3^; thus, surveillance for SAB and birth outcomes after 9vHPV vaccine exposures in pregnancy is needed. Using a multisite retrospective cohort, we conducted the current study to evaluate the associations between 9vHPV vaccine exposures during pregnancy or peripregnancy and selected pregnancy and birth outcomes (SABs, preterm births, small-for-gestational age [SGA] births, and major structural birth defects).

## Methods

### Cohort

The Vaccine Safety Datalink (VSD), a collaboration between the Centers for Disease Control and Prevention and several integrated health systems, includes electronic health data and detailed clinical records for approximately 3% of the US population.^21^ For this study, we analyzed data contributed by 7 participating VSD sites (Kaiser Permanente Northern California, Kaiser Permanente Southern California, Kaiser Permanente Northwest, Kaiser Permanente Washington, Kaiser Permanente Colorado, Marshfield Clinic, and HealthPartners). This study was approved by the institutional review boards of all 7 sites, which waived the informed consent requirement because this observational study posed minimal risk. We followed the Strengthening the Reporting of Observational Studies in Epidemiology (STROBE) reporting guideline.

Using a validated automated pregnancy algorithm,^22^ which was adapted for *International Statistical Classification of Diseases, Tenth Revision, Clinical Modification* and applied to claims, electronic health records (EHRs), and birth records, we identified pregnancies among girls and women aged 12 to 28 years (1) whose pregnancy ended between October 26, 2015, and November 15, 2018; (2) who had continuous insurance enrollment from 8 months before pregnancy start through 8 weeks after pregnancy end; and (3) who received a 4vHPV or 9VHPV vaccine either during pregnancy or in the 12 months before their last menstrual period (LMP). The algorithm applied a hierarchical approach to identify the pregnancy outcome, outcome date, gestational age at outcome, and pregnancy start date. Singleton pregnancies that ended in a live birth, stillbirth (fetal demise at 20 weeks’ gestation or later), or SAB (fetal demise before 20 weeks’ gestation) were included.^14,23^

For pregnancies that were identified by the pregnancy algorithm as SABs, clinical data, including LMP dates, estimated delivery dates, human chorionic gonadotropic testing results, and ultrasonography results, were manually reviewed and then entered into structured REDCap forms (Vanderbilt University) by trained medical record abstractors.^15,24^ Redacted ultrasonography and pathology reports were uploaded into REDCap. Confirmation and dating of SABs were assigned after adjudication by 2 of our physician investigators (E.O.K. and M.B.D.) and after secondary review of complex cases by 3 of our obstetric investigators (H.S.L., S.S.S., and V.G.). Medical record abstractors and adjudicators were blinded to the timing of vaccine exposures. Final classification and dating of SABs were consistent with that in previous work and based on published guidelines from the American College of Obstetricians and Gynecologists.^15,25^ Spontaneous abortions that were estimated to occur between 6 and 19 completed weeks of gestation were potentially eligible for inclusion.

For pregnancies that were identified through the pregnancy algorithm as stillbirths, the outcomes were confirmed through medical record review. The data were entered into structured REDCap forms, and gestational age was based on gestational age at delivery or the estimated delivery date.^15^

We excluded pregnancies that were classified as SABs before 6 weeks’ gestation, therapeutic abortions, ectopic pregnancies, multiple gestation pregnancies, and gestational trophoblastic disease. We also excluded girls and women with a prescription for an abortifacient or teratogenic medication during pregnancy or in the 8 months before pregnancy (eTable in the Supplement). In addition, we excluded girls and women with no outpatient visits within a VSD health system.

Pregnancies that ended in a live birth were linked with infant birth data. Gestational age was required to evaluate preterm birth. Birth weight and gestational age were required to evaluate SGA birth. For evaluation of major structural birth defects, at least 1 outpatient visit and 4 months of insurance enrollment in the first year were required for infants who survived their first year of life. We excluded infants with chromosomal disorders.

Covariates, such as race/ethnicity, comorbidities, health care utilization before and during pregnancy, and history of smoking, were assessed from automated EHR data. For pregnancies that ended in a SAB or stillbirth, smoking status, pregestational body mass index, and previous obstetric history were also collected during medical record review.

### Vaccine

**Figure 1.**
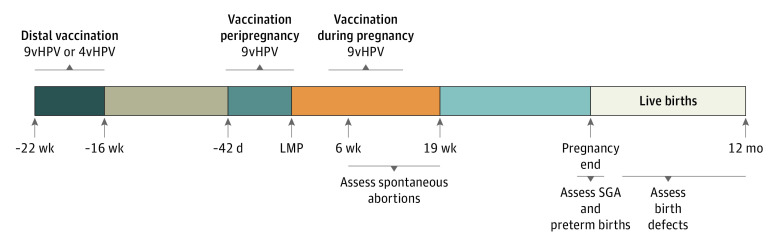
Vaccine Exposure Windows and Periods for Outcome Assessment 4vHPV indicates quadrivalent human papillomavirus vaccine; 9vHPV, 9-valent human papillomavirus vaccine; LMP, last menstrual period; and SGA, small for gestational age.

Human papillomavirus vaccine data came from VSD files, which captured vaccines recorded in the site EHR and medical or pharmacy claims and through bidirectional communication with state immunization registries. In the final analyses, we included women with the following vaccine exposure windows: distal exposure consisted of 9vHPV or 4vHPV vaccine administered from 22 to 16 weeks before LMP, peripregnancy consisted of 9vHPV vaccine administered from 42 days before LMP until LMP, and during pregnancy consisted of 9vHPV vaccine administered from LMP to 19 completed weeks’ gestation (Figure 1).

The distal exposure window included the 9vHPV or 4vHPV vaccine to limit exclusions on the basis of site-based variability in transition from 4vHPV to 9vHPV vaccines. In addition, given that pregnancies were initially identified according to pregnancy end dates, the inclusion of 4vHPV or 9vHPV vaccines in the distal exposure window reduced the potential differential inclusion of pregnancies with a shorter gestation. Girls and women with more than 1 eligible 9vHPV vaccine dose were assigned to a single exposure window, with during-pregnancy exposure being the highest priority and distal exposure being the lowest priority.

### Outcomes

We initially identified SABs through the automated pregnancy algorithm and assigned SABs as occurring at 10 weeks’ gestation. If estimated delivery date or LMP data were available in the algorithm, the information was used to recalculate the gestational age at SAB. On the basis of the outcome date and gestational age from the pregnancy algorithm, we reviewed the medical records for all SABs in female individuals who received an HPV vaccine in the following expanded exposure windows: expanded distal consisted of 4vHPV or 9vHPV vaccine administered from 24 to 14 weeks before LMP, expanded peripregnancy consisted of 9vHPV vaccine administered from 56 days before LMP until LMP, and all potential during-pregnancy exposures consisted of 9vHPV vaccine administered from LMP to 19 completed weeks’ gestation. The expanded exposure windows were used to select potentially eligible cases for medical record review, given that both the SAB outcome date and the gestational age at SAB could change after clinical adjudication, while limiting the overall medical record review burden.

As described, the physician or obstetric adjudicators among us, who were blinded to exposure status, assigned final outcomes (SAB or other birth outcome), SAB outcome date (earliest date of pregnancy loss after confirmation of an intrauterine pregnancy), and gestational age at SAB (based on ultrasonography measurements of fetal pole, yolk sac, or gestational sac).^26^ Cases with discrepant dating between sources (eg, serial ultrasonography, LMP, estimated delivery date), or where viability was unclear, underwent obstetric adjudication. Ten percent of medical records were adjudicated by both physician adjudicators, and agreement on pregnancy outcome was assessed by the κ statistic. For SABs, we assessed agreement on gestational age (within 7 days) and date of fetal demise (within 7 days).

Preterm birth was defined as a live birth before 37 weeks’ gestation according to clinical assessment and was obtained from the EHR or birth records. Small-for-gestational age birth, defined as below the 10th percentile, was calculated according to reference values described by Talge et al.^27^ Birth weights came from birth records or the EHR.

Major structural birth defects were identified using validated, defect-specific algorithms. These algorithms were originally developed for use with *International Classification of Diseases, Ninth Revision, Clinical Modification* codes^28^ and were adapted and validated for *International Statistical Classification of Diseases, Tenth Revision, Clinical Modification* codes.^29^

### Statistical Analysis

Baseline characteristics by HPV vaccine exposure window were explored using descriptive statistics. The primary comparisons were (1) girls and women with 9vHPV vaccine exposure during pregnancy vs those with 4vHPV or 9vHPV distal vaccine exposures, (2) girls and women with 9vHPV vaccine exposure peripregnancy vs those with 4vHPV or 9vHPV distal vaccine exposures, and (3) girls and women with 9vHPV vaccine exposure during pregnancy or peripregnancy vs those with 4vHPV or 9vHPV distal vaccine exposure.

We estimated stabilized inverse probability weight (IPW) to balance the covariates. Age, race/ethnicity, hospitalization before pregnancy, history of smoking, and VSD site were included in the propensity score. Six propensity scores were constructed (3 for the full cohort and 3 for pregnancies that ended in live births) and for the following contrasts: 9vHPV vaccine exposure during pregnancy vs 9vHPV or 4vHPV distal vaccine exposure, 9vHPV vaccine exposure peripregnancy vs 9vHPV or 4vHPV vaccine distal exposure, and 9vHPV vaccine exposure peripregnancy or during pregnancy vs 9vHPV or 4vHPV distal vaccine exposure. We evaluated whether the distribution of the propensity score overlapped between exposure groups and whether, after the IPW was applied, the covariates were better balanced between exposure groups by plotting standardized differences. We used time-dependent covariate Cox proportional hazards regression models to account for immortal time bias^30,31^ in evaluating 9vHPV vaccine exposure and SAB, and we used Poisson regression for other birth and infant outcomes, with IPWs applied to models. Associations are reported as hazard ratios (HRs), relative risks (RRs), or prevalence ratios (PRs) with 95% CIs.

Assuming a power of 80%, a statistical significance level of α = .05 with a 2-sided tail, 400 girls and women with during-pregnancy exposure and 400 with distal vaccine exposure, and an SAB rate of 14 per 100 pregnancies, this study was powered to detect an HR of 1.5 (8 additional SAB cases per 100 pregnancies). For preterm delivery and SGA, this study was powered to detect an RR of 1.75 (7.5 additional events per 100 births), assuming a 10% event rate. For birth defects, this study was powered to detect a PR of 4.0 (4.7 additional birth defects per 100 births). All analyses were performed in SAS/STAT, version 9.4 (SAS Institute, Inc.).

## Results

**Figure 2.**
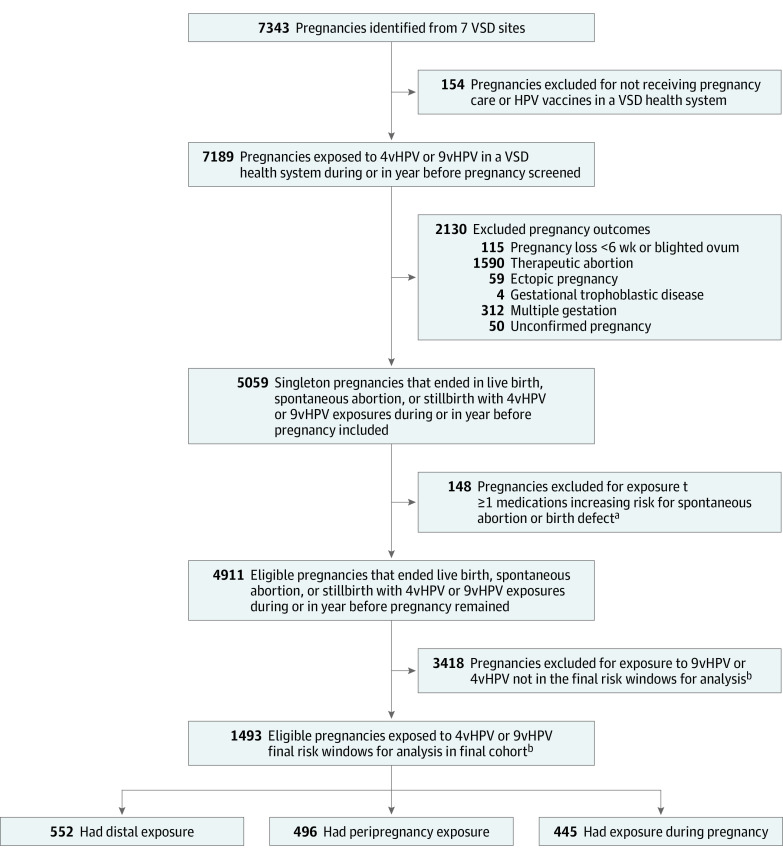
Flowchart of Pregnancies and Final Cohort Across 7 Vaccine Safety Datalink (VSD) Sites ^a^Medications that were excluded if dispensed 6 months before last menstrual period (LMP) through the end of pregnancy were prostaglandin analogs, vitamin A analogs, selected immunosuppressants, selected anticonvulsants, amiodarone hydrochloride, warfarin sodium, and lithium carbonate. ^b^The final exposure windows for analysis were as follows: distal exposure consisted of 9-valent human papillomavirus vaccine (9vHPV) or quadrivalent human papillomavirus vaccine (4vHPV) administered from 22 to 16 weeks before LMP, peripregnancy exposure consisted of 9vHPV administered from 42 days before LMP until LMP, and during-pregnancy exposure consisted of 9vHPV administered from LMP to 19 completed weeks’ gestation. For pregnancies in the distal exposure, 103 (18.7%) received 4vHPV and 449 (81.3%) received 9vHPV.

Across 7 VSD sites, we identified 7343 pregnancies that ended between October 26, 2015, and November 15, 2018, among girls and women with continuous insurance enrollment and 1 or more 4vHPV or 9vHPV vaccine exposures from 12 months before LMP through 20 weeks after LMP. We excluded 154 pregnancies (2.1%) because they received no pregnancy-related care or did not receive an HPV vaccine in the VSD health system. From the 7189 pregnancies remaining, we excluded 2130 (29.6%) because of a noneligible pregnancy outcome, 148 (2.1%) because of exposure to a teratogenic or abortifacient medication, and 3418 (47.5%) because their vaccine exposure did not occur during the pregnancy, peripregnancy, or in the distal window (Figure 2).

Of the 166 possible SABs from automated data, 80 (48.2%) remained in the cohort after medical record review and adjudication, 25 (15.0%) had 9vHPV vaccine exposures during pregnancy, 22 (13.3%) had 9vHPV vaccine exposure peripregnancy, and 33 (19.9%) had 4vHPV or 9vHPV distal vaccine exposures. The median (range) gestational age at SAB was 10 weeks and 3 days (6 weeks and 4 days to 18 weeks and 4 days). Sixteen cases (9.6%) underwent independent adjudication by 2 physicians, with substantial agreement in outcomes (κ = 0.75; 95% CI, 0.44-1.0). There was agreement on gestational age for all 7 SAB cases, and agreement on date of fetal demise for 6 cases.

The final cohort for analysis of SAB comprised 1493 pregnancies in women with a mean (SD) maternal age of 23.9 (2.9) years. Of these pregnancies, 445 (29.8%) had 9vHPV vaccine exposure during pregnancy, 496 (33.2%) had 9vHPV vaccine exposure peripregnancy, and 552 (37.0%) had 4vHPV or 9vHPV distal exposures (with 449 [81.3%] receiving 9vHPV vaccine and 103 [18.7%] receiving 4vHPV vaccine). Compared with those with distal exposures, girls and women with vaccine exposures during pregnancy were slightly younger (mean [SD] age, 23.5 [2.9] years vs 24.1 [2.9] years) and were more likely to have received a first 9vHPV vaccine dose (198 [44.5%] vs 208 [37.7%]). Additional baseline characteristics by vaccine exposure window are shown in Table 1. Performance of propensity score and IPW indicated adequate overlap of IPW across exposure groups and that IPW balanced the baseline characteristics across groups (eFigures 1 and 2 in the Supplement).

**Table 1.** Baseline Cohort Characteristics by Human Papillomavirus Vaccine Exposure Window^a^

Characteristic	No. (%)		
	9vHPV or 4vHPV vaccine distal exposure (n = 552)	9vHPV vaccine exposure	
		Peripregnancy (n = 496)	During pregnancy (n = 445)
Maternal age, mean (SD), y	24.1 (2.9)	24.0 (2.7)	23.5 (2.9)^b^
Maternal age at end of pregnancy, y			
12-15	<5 (<1)^c^	<5 (<1)^c^	6 (1.4)
16-19	51 (9.2)	33 (6.7)	49 (11.0)
20-24	196 (35.5)	205 (41.3)	188 (42.3)
25-28	303 (54.9)	256 (51.6)	202 (53.4)
Maternal race/ethnicity			
Asian	44 (8.0)	40 (8.1)	30 (6.7)
African American, non-Hispanic	46 (8.3)	39 (7.9)	44 (9.9)
Hispanic	342 (62.0)	312 (62.9)	281 (63.2)
White, non-Hispanic	87 (15.8)	79 (15.9)	67 (15.1)
Other or not available	33 (6.0)	26 (5.2)	23 (5.2)
Poverty, mean (SD)^d^	28 (15)	27 (16)	27 (17)
Prenatal care	531 (96.2)	476 (96.0)	418 (93.9)
Gestational week at first prenatal visit, mean (SD)	7.1 (2.8)	7.1 (3.0)	7.2 (3.7)
HPV vaccine dose			
First	208 (37.7)	224 (45.2)^b^	198 (44.5)^b^
Second	231 (41.9)	150 (30.2)^b^	141 (31.7)^b^
Third	113 (20.5)	122 (24.6)^b^	106 (23.8)^b^
Receipt of other vaccines during pregnancy^e^	486 (93.1)	442 (89.1)	401 (91.5)
Receipt of live virus vaccine during pregnancy	<5 (<1)^c^	<5 (<1)^c^	8 (1.8)
History of ever smoking^f^	42 (7.6)	41 (8.3)	49 (11.0)
Obesity^g^	189 (34.2)	167 (33.7)	162 (36.4)
Prepregnancy diabetes	14 (2.5)	7 (1.4)	7 (1.6)
Systemic lupus erythematous	<5 (<1)^c^	0	<5 (<1)^c^
Hospitalization before pregnancy	45 (8.2)	52 (10.5)	57 (12.8)
Primigravida^h^	230 (43)	205 (42.0)	199 (46.0)

Abbreviations: 4vHPV, quadrivalent human papillomavirus vaccine; 9vHPV, 9-valent human papillomavirus vaccine; LMP, last menstrual period.

^a^ Exposure windows: distal consisted of 9vHPV or 4vHPV vaccine administered from 22 to 16 weeks before LMP, peripregnancy consisted of 9vHPV vaccine administered from 42 days before LMP until LMP, during pregnancy consisted of 9vHPV vaccine administered from LMP to 19 completed weeks’ gestation.

^b^ Denotes differences between girls and women in the peripregnancy or during-pregnancy exposure group and those in the distal exposure group, which differed significantly at *P* < .05.

^c^ Denotes cells with fewer than 5 participants.

^d^ Percentage in census tract at less than 150% federal poverty level.

^e^ Other vaccines included meningococcal, influenza, and acellular pertussis vaccines.

^f^ Smoking was assessed through electronic health record and medical record review.

^g^ Obesity was assessed through prepregnancy weight, diagnosis codes, and medical record review.

^h^ Primigravida was assessed through birth records and medical record review; values were missing for 34 pregnancies, 8 pregnancies in the distal exposure group, 7 pregnancies in the peripregnancy exposure group, and 5 pregnancies in the during-pregnancy exposure group.

The 9vHPV vaccine exposures during pregnancy were not associated with SAB compared with distal exposures (HR, 1.12; 95% CI, 0.66-1.93). Findings were similar for 9vHPV vaccine exposures peripregnancy (RR, 0.72; 95% CI, 0.42-1.24) and for 9vHPV vaccine exposures during pregnancy or peripregnancy (HR, 1.20; 95% CI, 0.77-1.88) compared with distal exposures (Table 2).

**Table 2.** Risks for Spontaneous Abortion (SAB), Preterm Birth, Small-for-Gestational Age Birth (SGA), and Major Structural Birth Defects by Human Papillomavirus Vaccine Exposure Window^a^

Variable	No. (%)			Risk of event		
	Distal exposure	Peripregnancy exposure	During-pregnancy exposure	Peripregnancy vs distal exposure	During pregnancy vs distal exposure	During pregnancy or peripregnancy vs distal exposure
Pregnancies, No.	552	496	445	NA	NA	NA
SAB	33 (6.0)	22 (4.4)	25 (5.6)	RR (95% CI): 0.72 (0.42-1.24)^b^	HR (95% CI): 1.12 (0.66-1.93)^c^	HR (95% CI): 1.20 (0.77-1.88)^c^
Live births with gestational age, No.	518	474	418	NA	NA	NA
Preterm births^d^	42 (8.1)	27 (5.7)	26 (6.2)	RR (95% CI): 0.72 (0.45-1.17)^c^	RR (95% CI): 0.73 (0.44-1.20)^c^	RR (95% CI): 0.75 (0.50-1.13)^c^
Live births with gestational age and birth weight, No.	425	388	341	NA	NA	NA
SGA births^e^	27 (6.4)	27 (7.0)	29 (8.5)	RR (95% CI): 1.10 (0.65-1.88)^c^	RR (95% CI): 1.31 (0.78-2.20)^c^	RR (95% CI): 1.18 (0.75-1.85)^c^
Live births with follow-up, No.^f^	414	363	320	NA	NA	NA
Major structural birth defects	4 (1.0)	4 (1.1)	4 (1.3)	PR (95% CI): 1.03 (0.26-4.07)^g^	PR (95% CI): 1.30 (0.36-4.69)^g^	PR (95% CI): 1.06 (0.34-3.33)^g^

Abbreviations: HR, hazard ratio; LMP, last menstrual period; NA, not applicable; PR, prevalence ratio; and RR, relative risk.

^a^ Exposure windows: distal consisted of 9vHPV or 4vHPV vaccine administered from 22 to 16 weeks before LMP, peripregnancy consisted of 9vHPV vaccine administered from 42 days before LMP until LMP, during pregnancy consisted of 9vHPV vaccine administered from LMP to 19 completed weeks’ gestation.

^b^ Using 9vHPV or 4vHPV vaccine, with inverse probability weights for age, race/ethnicity, hospitalization before pregnancy, smoking, and site.

^c^ Using 9vHPV or 4vHPV vaccine as a time-dependent exposure in a Cox proportional hazards regression model, with inverse probability weights for age, race/ethnicity, hospitalization before pregnancy, smoking, and site.

^d^ Preterm birth defined as live birth before 37 weeks’ gestation.

^e^ SGA birth defined as below 10th percentile, based on Talge et al.^27^

^f^ For evaluation of birth defect outcomes, infants who survived the first year were required to have at least 1 outpatient visit in the health system, 4 months of insurance in the first year of life, and no diagnosis of a chromosomal anomaly.

^g^ Using 9vHPV or 4vHPV vaccine, with inverse probability weights for age, race/ethnicity, hospitalization before pregnancy, smoking, and site.

Of the 1493 pregnancies in the cohort, 1409 (94.4%) ended in a live birth with gestational age data available. Preterm birth occurred in 26 pregnancies (6.2%) with during-pregnancy vaccine exposures, 27 (5.7%) with peripregnancy vaccine exposures, and 42 (8.1%) with distal exposures. Exposure to 9vHPV vaccine during pregnancy was not associated with preterm birth (RR, 0.73; 95% CI, 0.44-1.20). Results were similar regarding the association between peripregnancy vaccine exposures and preterm birth (RR, 0.72; 95% CI, 0.45-1.17) as well as between vaccine exposures during pregnancy or peripregnancy and preterm birth (RR, 0.75; 95% CI, 0.50-1.13) (Table 2).

Of the 1154 live births with data available for gestational age and birth weight, SGA births occurred in 29 pregnancies (8.5%) with 9vHPV vaccine exposure during pregnancy, 27 (7.0%) 9vHPV vaccine exposures peripregnancy, and 27 (6.4%) 4vHPV or 9vHPV distal exposures. In adjusted analyses, 9vHPV vaccine exposures in any exposure window (during pregnancy [RR, 1.31; 95% CI, 0.78-2.20], peripregnancy [RR, 1.10; 95% CI, 0.65-1.88], and during pregnancy or peripregnancy [RR, 1.18; 95% CI, 0.75-1.85]) were not associated with SGA birth (Table 2).

Of the 1097 live births with available infant follow-up, birth defects were rare in all exposure groups and were not associated with exposures during pregnancy (PR, 1.30; 95% CI, 0.36-4.69), peripregnancy (PR, 1.03; 95% CI, 0.26-4.07), or during pregnancy or peripregnancy (PR, 1.06; 95% CI, 0.34-3.33) (Table 2). A list of birth defects by exposure window is shown in Table 3.

**Table 3.** List of Major Structural Birth Defects by Human Papillomavirus Vaccine Exposure Window

9vHPV or 4vHPV distal exposure (n = 4)	9vHPV peripregnancy exposure (n = 4)	9vHPV during-pregnancy exposure (n = 4)
Ventricular septal defect	Ventricular septal defect	Ventricular septal defect (2 cases)
Microcephaly	Microcephaly	Microcephaly
Nasofrontal encephalocele, unilateral cleft lip with cleft palate, and ventricular septal defect	Cleft soft palate	Pyloric stenosis
Unilateral renal agenesis	Septo-optic dysplasia	NA

Abbreviations: 4vHPV, quadrivalent human papillomavirus vaccine; 9vHPV, 9-valent human papillomavirus vaccine; NA, not applicable.

## Discussion

In this multisite, observational retrospective cohort study, exposures to 9vHPV vaccine during or around the time of pregnancy were uncommon and were not associated with SAB or selected adverse birth outcomes. These findings substantially add to the literature on 9vHPV vaccine exposures in pregnancy^20^ and are consistent with the results of previous studies of 4vHPV vaccination in pregnancy.^14,15,32,33^ We believe that this research supports current recommendations by the Advisory Committee on Immunization Practices that 9vHPV vaccine, although not recommended for use during pregnancy, can be administered to female individuals of reproductive age without routine pregnancy testing.

In 4vHPV vaccine prelicensure trials, less than 1% of participants were vaccinated within 30 days of pregnancy and no increased risks for SAB or adverse birth outcomes were observed compared with those who received placebo.^33^ Additional data on 4vHPV vaccine exposures from the manufacturer’s postlicensure pregnancy registry indicated that, among 1752 prospective reports, the SAB rate was 6.7 per 100 pregnancies and the prevalence of birth defects was 2.4 per 100 live births, which were consistent with background rates.^18^ Scheller et al^16^ evaluated pregnancies in Denmark with 4vHPV vaccine exposures over a 7-year period that were propensity score–matched to unexposed pregnancies and found that 4vHPV was not associated with SABs, preterm births, SGA births, or birth defects. Other observational studies have also found no association between 4vHPV exposures during pregnancy and SAB or other adverse birth outcomes.^14,15,17,19^

To date, data on pregnancy or birth outcomes after 9vHPV vaccine exposures have been limited to passive reports and secondary analyses of prelicensure clinical trial data. In 2019, Landazabal et al^34^ described 82 reports to the Vaccine Adverse Event Reporting System of 9vHPV administration during pregnancy over a 3-year period. For 60 of the 82 reports, the only adverse event was that the vaccine was inadvertently administered during pregnancy. The study included 3 reports of SAB, 2 reports of vaginal bleeding, and no report of concerning safety signals.^34^ Moreira et al^20^ found from analyses of 7 phase 3 clinical trials that, because pregnancy tests were conducted before each vaccine dose, exposures during pregnancy were uncommon. Among approximately 170 women with the estimated date of conception within 30 days of vaccination, SAB occurred in 20% of women who received a 9vHPV vaccine vs 9.2% of those who received a 4vHPV vaccine.^20^ The lower SAB rate in the present study may reflect the differences in case ascertainment or case confirmation.^20^ Nevertheless, analyses in the present study did not support the increased risks for SAB after 9vHPV vaccine exposures during pregnancy compared with distal exposures.

In prelicensure trials, birth outcomes, including birth defects and premature births, did not differ between the 47 infants born to mothers with 9vHPV vaccine exposures vs 42 infants born to mothers with 4vHPV vaccine exposures.^20^ The data we used included more than 800 live births with during-pregnancy or peripregnancy 9vHPV vaccine exposures and nearly 700 live births with follow-up to evaluate for birth defects.

To our knowledge, this study is the first postlicensure study to systematically describe adverse pregnancy and birth outcomes after 9vHPV vaccine exposures during or around the time of pregnancy. Given the continual need for catch-up 9vHPV vaccination in women up to age 26 years, along with the expanded use of 9vHPV vaccination in women up to age 45 years, the findings from this study can inform counseling after inadvertent 9vHPV vaccine exposures during or around the time of pregnancy.

### Limitations

This study has several limitations. First, as an observational study, women vaccinated during pregnancy or peripregnancy may have differed from those vaccinated in the distal exposure window in ways that were associated with risks for SAB or adverse birth outcomes. We were only able to adjust for available covariates. For pregnancies that ended in live births, some covariates were available only through automated data, whereas for pregnancies that ended in stillbirths or SABs, the covariates were also collected through medical record review. Differential ascertainment and unmeasured confounding may have been an issue. Second, many potential SABs were excluded after medical record review and adjudication because an intrauterine pregnancy could not be confirmed or the pregnancy did not reach 6 weeks’ gestation. Inclusion of these pregnancies would have increased the sample size but also increased uncertainty regarding the outcome and date of fetal demise. Third, consistent with previous work and best practices in maternal pharmacoepidemiology, the cohort in this study was limited to girls and women with continuous insurance enrollment and who had received at least 1 HPV vaccine dose within 1 year of a pregnancy and, for the assessment of birth defects, infants with 4 months of insurance enrollment.^14,15,35^ This approach was necessary to ensure the capture of vaccine exposures and birth defect outcomes as well as to increase comparability across groups, but it may have decreased generalizability. Fourth, given the infrequency of inadvertent pregnancy exposures, this study was not powered to evaluate major structural birth defects or to identify modest increases after 9vHPV vaccine exposure for the remaining pregnancy outcomes.

## Conclusions

This observational cohort study found that exposures to 9vHPV vaccine during or around the time of pregnancy were uncommon and not associated with SAB or other adverse birth outcomes. These results can inform patient counseling after inadvertent 9vHPV during-pregnancy or peripregnancy HPV vaccine exposures.

## Notes

Supplement. **eFigure 1.** Histograms of Propensity Scores for Full Cohort **eFigure 2.** Standardized Differences Before and After IPW for Selected Risk Factors, Full Cohort **eTable.** List of Teratogenic and Abortifacient Medication Exclusions if Prescription Fill During Pregnancy or Within 8 Months of Last Menstrual Period

## References

[1] Markowitz LE, Dunne EF, Saraiya M, Lawson HW, Chesson H, Unger ER; Centers for Disease Control and Prevention (CDC); Advisory Committee on Immunization Practices (ACIP). Quadrivalent human papillomavirus vaccine: recommendations of the Advisory Committee on Immunization Practices (ACIP). MMWR Recomm Rep. 2007;56(RR-2):1-24. 17380109

[2] Petrosky E, Bocchini JA Jr, Hariri S, ; Centers for Disease Control and Prevention (CDC). Use of 9-valent human papillomavirus (HPV) vaccine: updated HPV vaccination recommendations of the advisory committee on immunization practices. MMWR Morb Mortal Wkly Rep. 2015;64(11):300-304. 25811679 PMC4584883

[3] Markowitz LE, Gee J, Chesson H, Stokley S. Ten years of human papillomavirus vaccination in the United States. Acad Pediatr. 2018;18(2S):S3-S10. doi:10.1016/j.acap.2017.09.014 29502635 PMC11331487

[4] Joura EA, Giuliano AR, Iversen OE, ; Broad Spectrum HPV Vaccine Study. A 9-valent HPV vaccine against infection and intraepithelial neoplasia in women. N Engl J Med. 2015;372(8):711-723. doi:10.1056/NEJMoa1405044 25693011

[5] Huh WK, Joura EA, Giuliano AR, . Final efficacy, immunogenicity, and safety analyses of a nine-valent human papillomavirus vaccine in women aged 16-26 years: a randomised, double-blind trial. Lancet. 2017;390(10108):2143-2159. doi:10.1016/S0140-6736(17)31821-4 28886907

[6] Palefsky JM, Giuliano AR, Goldstone S, . HPV vaccine against anal HPV infection and anal intraepithelial neoplasia. N Engl J Med. 2011;365(17):1576-1585. doi:10.1056/NEJMoa1010971 22029979

[7] Giuliano AR, Palefsky JM, Goldstone S, . Efficacy of quadrivalent HPV vaccine against HPV infection and disease in males. N Engl J Med. 2011;364(5):401-411. doi:10.1056/NEJMoa0909537 21288094 PMC3495065

[8] Markowitz LE, Liu G, Hariri S, Steinau M, Dunne EF, Unger ER. Prevalence of HPV after introduction of the vaccination program in the United States. Pediatrics. 2016;137(3):e20151968. doi:10.1542/peds.2015-1968 26908697

[9] Hariri S, Bennett NM, Niccolai LM, ; HPV-IMPACT Working Group. Reduction in HPV 16/18-associated high grade cervical lesions following HPV vaccine introduction in the United States: 2008-2012. Vaccine. 2015;33(13):1608-1613. doi:10.1016/j.vaccine.2015.01.084 25681664 PMC7522784

[10] Spinner C, Ding L, Bernstein DI, . Human papillomavirus vaccine effectiveness and herd protection in young women. Pediatrics. 2019;143(2):e20181902. doi:10.1542/peds.2018-1902 30670582 PMC6361347

[11] Kahn JA, Widdice LE, Ding L, . Substantial decline in vaccine-type human papillomavirus (HPV) among vaccinated young women during the first 8 years after HPV vaccine introduction in a community. Clin Infect Dis. 2016;63(10):1281-1287. doi:10.1093/cid/ciw533 27655996 PMC5091346

[12] Elam-Evans LD, Yankey D, Singleton JA, . National, regional, state, and selected local area vaccination coverage among adolescents aged 13-17 years: United States, 2019. MMWR Morb Mortal Wkly Rep. 2020;69(33):1109-1116. doi:10.15585/mmwr.mm6933a1 32817598 PMC7439984

[13] Meites E, Szilagyi PG, Chesson HW, Unger ER, Romero JR, Markowitz LE. Human papillomavirus vaccination for adults: updated recommendations of the Advisory Committee on Immunization Practices. MMWR Morb Mortal Wkly Rep. 2019;68(32):698-702. doi:10.15585/mmwr.mm6832a3 31415491 PMC6818701

[14] Lipkind HS, Vazquez-Benitez G, Nordin JD, . Maternal and infant outcomes after human papillomavirus vaccination in the periconceptional period or during pregnancy. Obstet Gynecol. 2017;130(3):599-608. doi:10.1097/AOG.0000000000002191 28796684 PMC6496947

[15] Kharbanda EO, Vazquez-Benitez G, Lipkind HS, . Risk of spontaneous abortion after inadvertent human papillomavirus vaccination in pregnancy. Obstet Gynecol. 2018;132(1):35-44. doi:10.1097/AOG.0000000000002694 29889760 PMC6019196

[16] Scheller NM, Pasternak B, Mølgaard-Nielsen D, Svanström H, Hviid A. Quadrivalent HPV vaccination and the risk of adverse pregnancy outcomes. N Engl J Med. 2017;376(13):1223-1233. doi:10.1056/NEJMoa1612296 28355499

[17] Faber MT, Duun-Henriksen AK, Dehlendorff C, Tatla MK, Munk C, Kjaer SK. Adverse pregnancy outcomes and infant mortality after quadrivalent HPV vaccination during pregnancy. Vaccine. 2019;37(2):265-271. doi:10.1016/j.vaccine.2018.11.030 30503078

[18] Goss MA, Lievano F, Buchanan KM, Seminack MM, Cunningham ML, Dana A. Final report on exposure during pregnancy from a pregnancy registry for quadrivalent human papillomavirus vaccine. Vaccine. 2015;33(29):3422-3428. doi:10.1016/j.vaccine.2015.04.014 25869893

[19] Sy LS, Meyer KI, Klein NP, . Postlicensure safety surveillance of congenital anomaly and miscarriage among pregnancies exposed to quadrivalent human papillomavirus vaccine. Hum Vaccin Immunother. 2018;14(2):412-419. doi:10.1080/21645515.2017.1403702 29140750 PMC5806640

[20] Moreira ED Jr, Block SL, Ferris D, . Safety profile of the 9-valent HPV vaccine: a combined analysis of 7 phase III clinical trials. Pediatrics. 2016;138(2):e20154387. doi:10.1542/peds.2015-4387 27422279

[21] Baggs J, Gee J, Lewis E, . The Vaccine Safety Datalink: a model for monitoring immunization safety. Pediatrics. 2011;127(suppl 1):S45-S53. doi:10.1542/peds.2010-1722H 21502240

[22] Naleway AL, Gold R, Kurosky S, . Identifying pregnancy episodes, outcomes, and mother-infant pairs in the Vaccine Safety Datalink. Vaccine. 2013;31(27):2898-2903. doi:10.1016/j.vaccine.2013.03.069 23639917

[23] Kharbanda EO, Vazquez-Benitez G, Lipkind HS, . Evaluation of the association of maternal pertussis vaccination with obstetric events and birth outcomes. JAMA. 2014;312(18):1897-1904. doi:10.1001/jama.2014.14825 25387187 PMC6599584

[24] Harris PA, Taylor R, Thielke R, Payne J, Gonzalez N, Conde JG. Research electronic data capture (REDCap)—a metadata-driven methodology and workflow process for providing translational research informatics support. J Biomed Inform. 2009;42(2):377-381. doi:10.1016/j.jbi.2008.08.010 18929686 PMC2700030

[25] The American College of Obstetricians and Gynecologists Practice Bulletin no. 150. Early pregnancy loss. Obstet Gynecol. 2015;125(5):1258-1267. doi:10.1097/01.AOG.0000465191.27155.25 25932865

[26] Papaioannou GI, Syngelaki A, Poon LC, Ross JA, Nicolaides KH. Normal ranges of embryonic length, embryonic heart rate, gestational sac diameter and yolk sac diameter at 6-10 weeks. Fetal Diagn Ther. 2010;28(4):207-219. doi:10.1159/000319589 20847544

[27] Talge NM, Mudd LM, Sikorskii A, Basso O. United States birth weight reference corrected for implausible gestational age estimates. Pediatrics. 2014;133(5):844-853. doi:10.1542/peds.2013-3285 24777216

[28] Kharbanda EO, Vazquez-Benitez G, Romitti PA, . Identifying birth defects in automated data sources in the Vaccine Safety Datalink. Pharmacoepidemiol Drug Saf. 2017;26(4):412-420. doi:10.1002/pds.4153 28054412 PMC6506837

[29] Kharbanda EO, Vazquez-Benitez G, DeSilva MB, . Developing algorithms for identifying major structural birth defects using automated electronic health data. Pharmacoepidemiol Drug Saf. 2021;30(2):266-274. doi:10.1002/pds.5177 33219586 PMC9116134

[30] Vazquez-Benitez G, Kharbanda EO, Naleway AL, . Risk of preterm or small-for-gestational-age birth after influenza vaccination during pregnancy: caveats when conducting retrospective observational studies. Am J Epidemiol. 2016;184(3):176-186. doi:10.1093/aje/kww043 27449414 PMC5003116

[31] Daniel S, Koren G, Lunenfeld E, Levy A. Immortal time bias in drug safety cohort studies: spontaneous abortion following nonsteroidal antiinflammatory drug exposure. Am J Obstet Gynecol. 2015;212(3):307.e1-307.e6. doi:10.1016/j.ajog.2014.09.028 25265406

[32] Vichnin M, Bonanni P, Klein NP, . An overview of quadrivalent human papillomavirus vaccine safety: 2006 to 2015. Pediatr Infect Dis J. 2015;34(9):983-991. doi:10.1097/INF.0000000000000793 26107345

[33] Garland SM, Ault KA, Gall SA, ; Quadrivalent Human Papillomavirus Vaccine Phase III Investigators. Pregnancy and infant outcomes in the clinical trials of a human papillomavirus type 6/11/16/18 vaccine: a combined analysis of five randomized controlled trials. Obstet Gynecol. 2009;114(6):1179-1188. doi:10.1097/AOG.0b013e3181c2ca21 19935017

[34] Landazabal CS, Moro PL, Lewis P, Omer SB. Safety of 9-valent human papillomavirus vaccine administration among pregnant women: adverse event reports in the Vaccine Adverse Event Reporting System (VAERS), 2014-2017. Vaccine. 2019;37(9):1229-1234. doi:10.1016/j.vaccine.2018.11.077 30660400 PMC6505695

[35] Huybrechts KF, Bateman BT, Hernández-Díaz S. Use of real-world evidence from healthcare utilization data to evaluate drug safety during pregnancy. Pharmacoepidemiol Drug Saf. 2019;28(7):906-922. doi:10.1002/pds.4789 31074570 PMC6823105

