# Supplementary material for: Association of Inadvertent 9-Valent Human Papillomavirus Vaccine in Pregnancy With Spontaneous Abortion and Adverse Birth Outcomes
Source: JAMA Netw Open. 2021 Apr 5;4(4):e214340. doi: 10.1001/jamanetworkopen.2021.4340 (PMC8022219; doi:10.1001/jamanetworkopen.2021.4340)
Supplement: Supplement. — eFigure 1. Histograms of Propensity Scores for Full Cohort eFigure 2. Standardized Differences Before and After IPW for Selected Risk Factors, Full Cohort eTable. List of Teratogenic and Abortifacient Medication Exclusions if Prescription Fill During Pregnancy or Within 8 Months of Last Menstrual Period [file jamanetwopen-e214340-s001.pdf]

Supplementary Online Content

Kharbanda EO, Vazquez-Benitez G, DeSilva MB, et al. Association of inadvertent 9-valent human papillomavirus vaccine in pregnancy with spontaneous abortion and adverse birth outcomes. *JAMA Netw Open*. 2021;4(4):e214340. doi:10.1001/jamanetworkopen.2021.4340

eFigure 1. Histograms of Propensity Scores for Full Cohort

eFigure 2. Standardized Differences Before and After IPW for Selected Risk Factors, Full Cohort

eTable. List of Teratogenic and Abortifacient Medication Exclusions if Prescription Fill During Pregnancy or Within 8 Months of Last Menstrual Period

This supplementary material has been provided by the authors to give readers additional information about their work.

© 2021 Kharbanda EO et al. *JAMA Network Open.*

eFigure 1. Histograms of Propensity Scores for Full Cohort

a) During pregnancy vs Distal

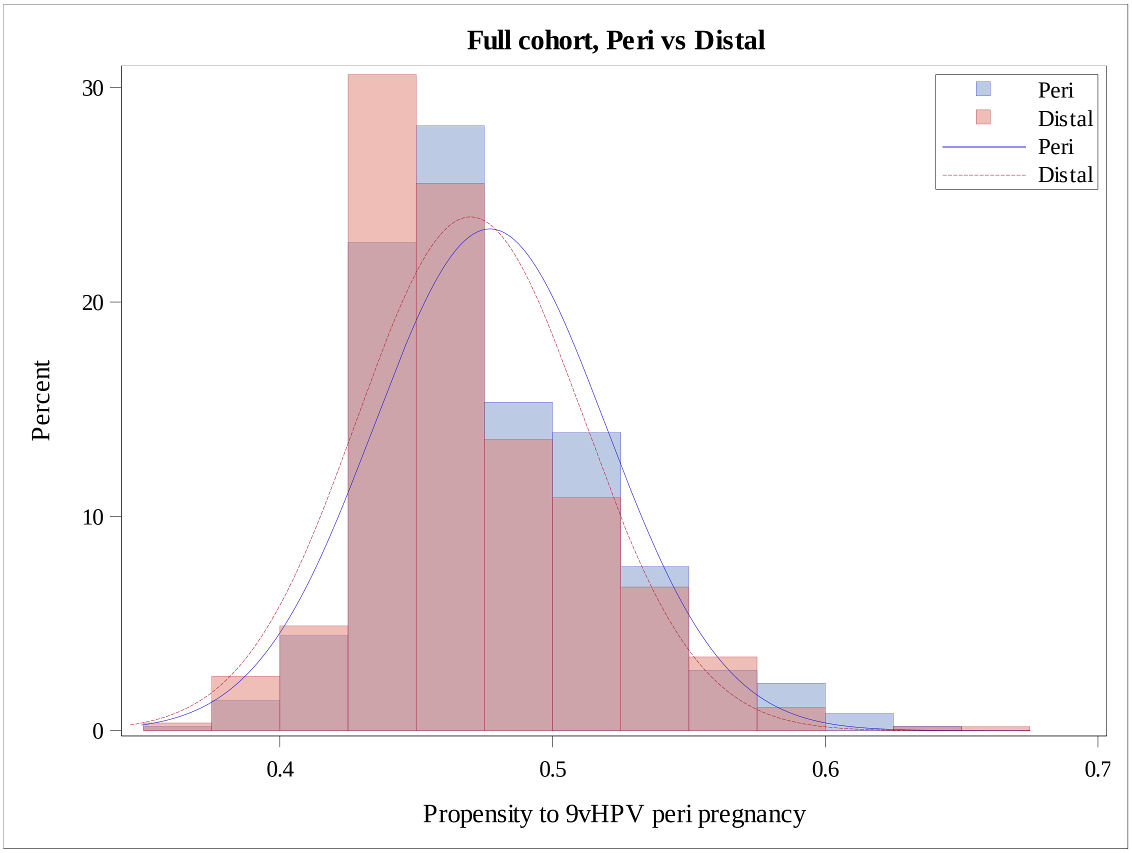

© 2021 Kharbanda EO et al. *JAMA Network Open*

b) Peri-pregnancy vs Distal

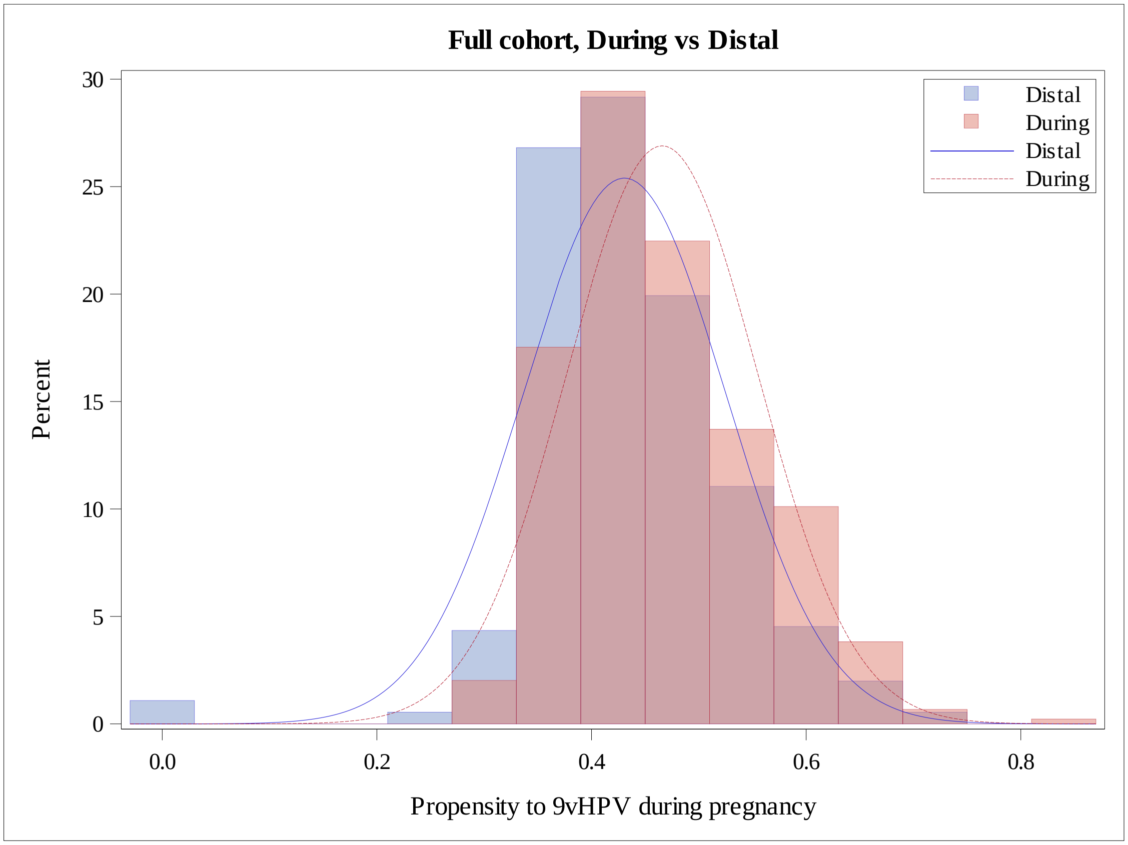

© 2021 Kharbanda EO et al. *JAMA Network Open*

c) Peri-pregnancy or During pregnancy vs Distal, for live birth cohort

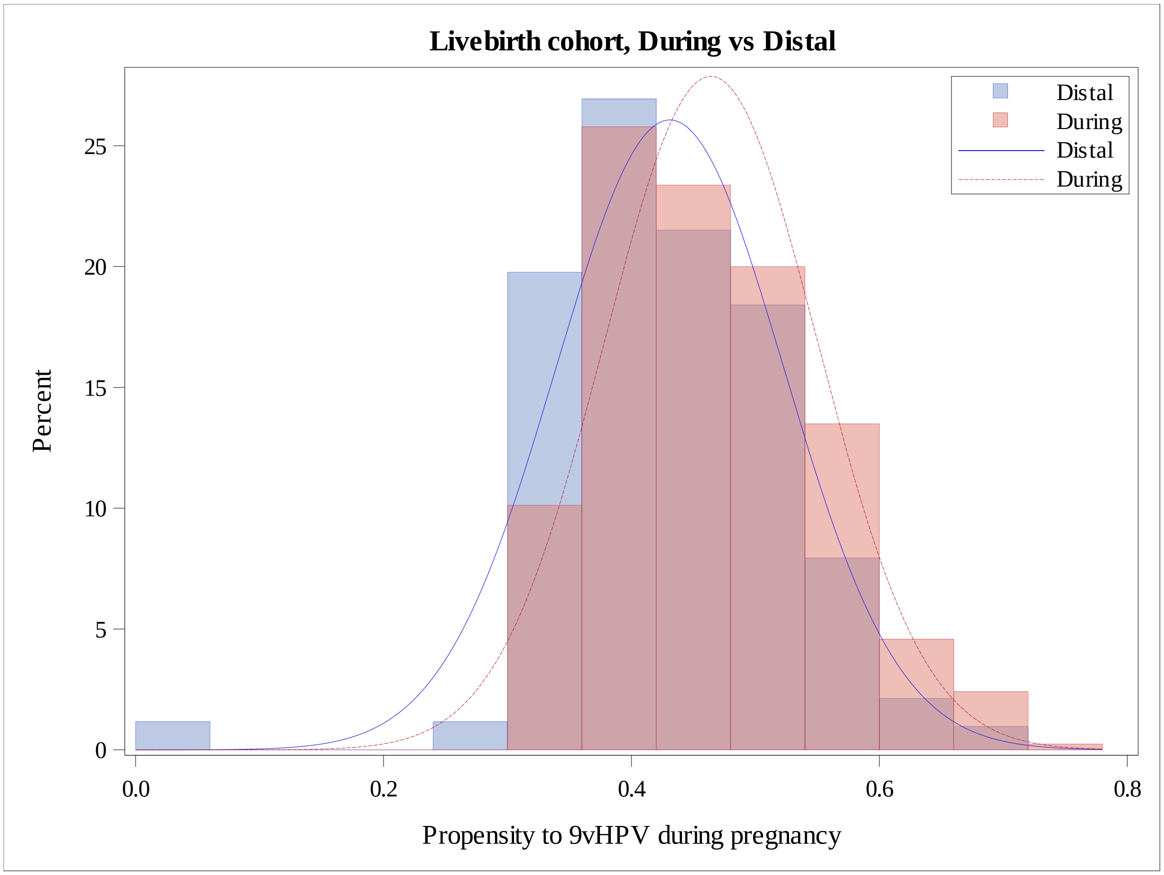

© 2021 Kharbanda EO et al. *JAMA Network Open*

d) During pregnancy vs Distal

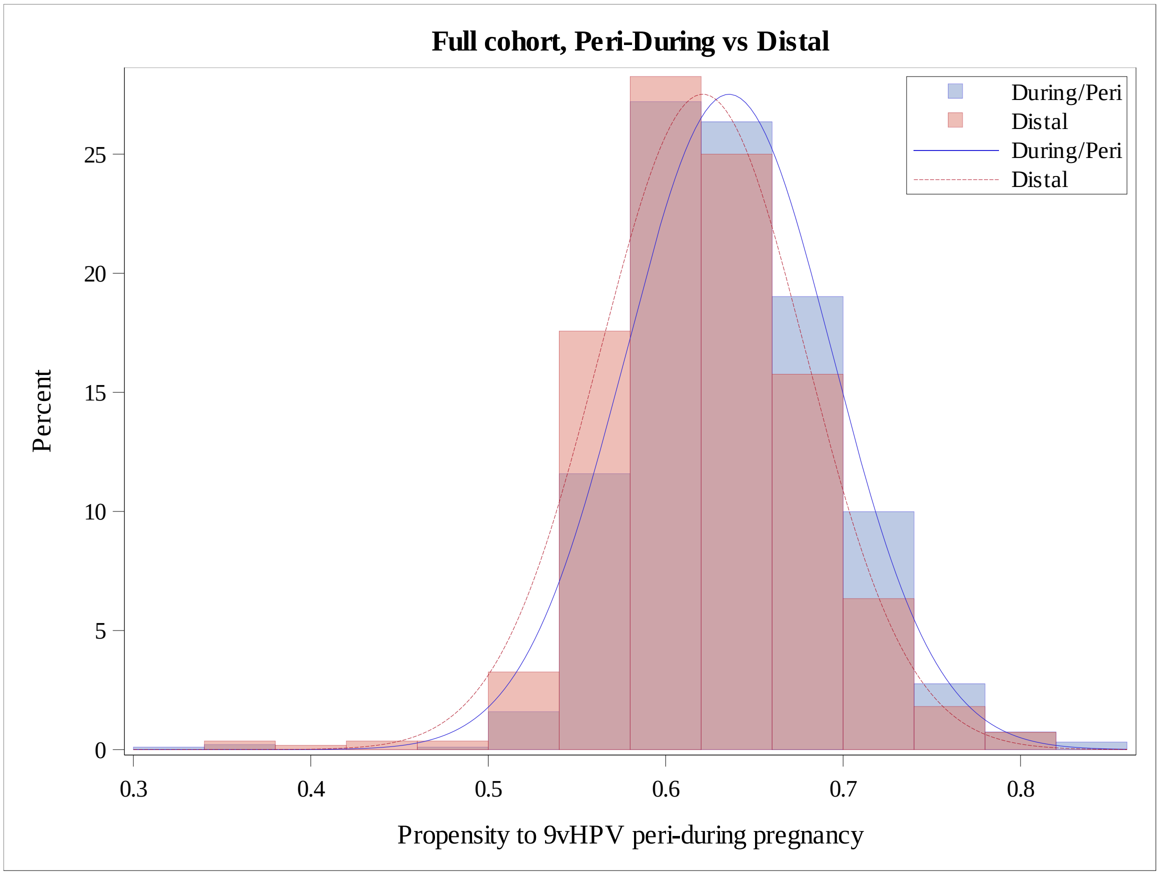

© 2021 Kharbanda EO et al. *JAMA Network Open*

e) Peri-pregnancy vs Distal

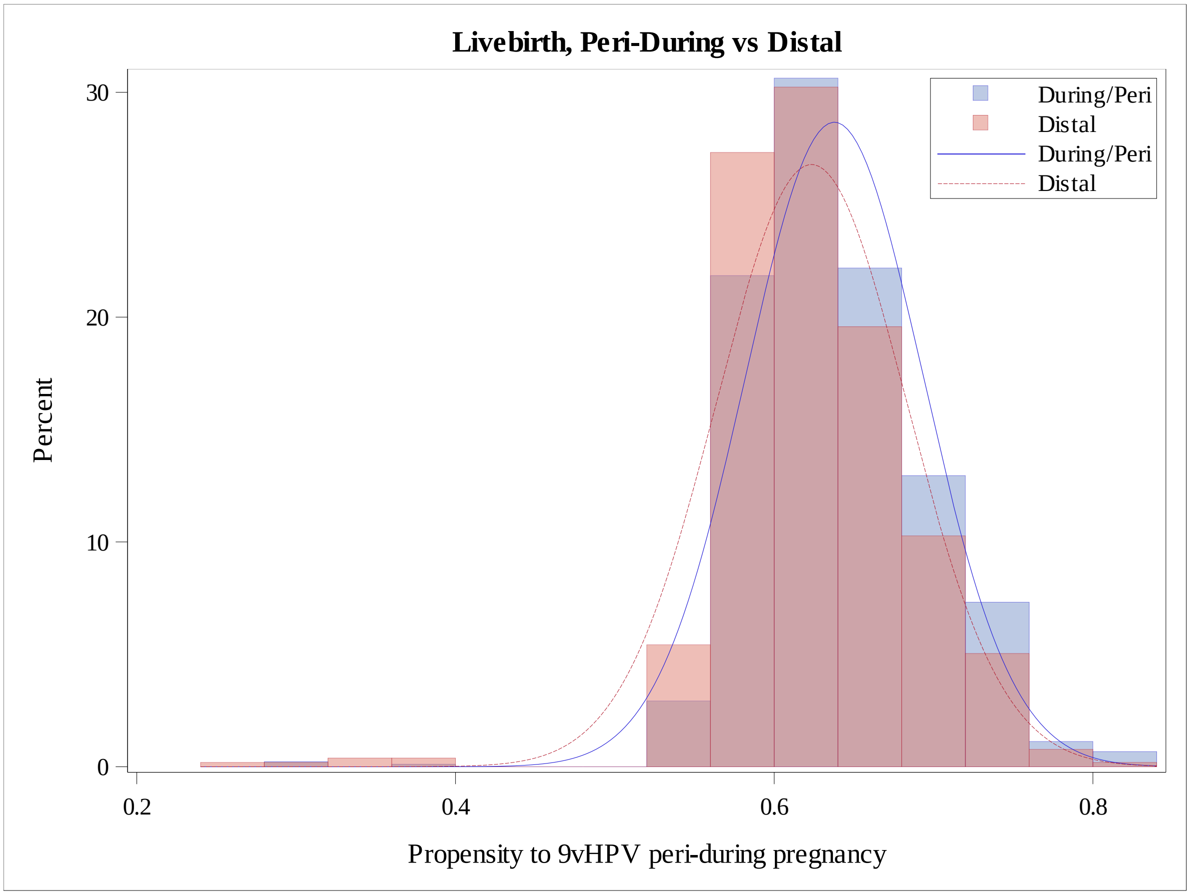

© 2021 Kharbanda EO et al. *JAMA Network Open*

f) Peri-pregnancy or During pregnancy vs Distal

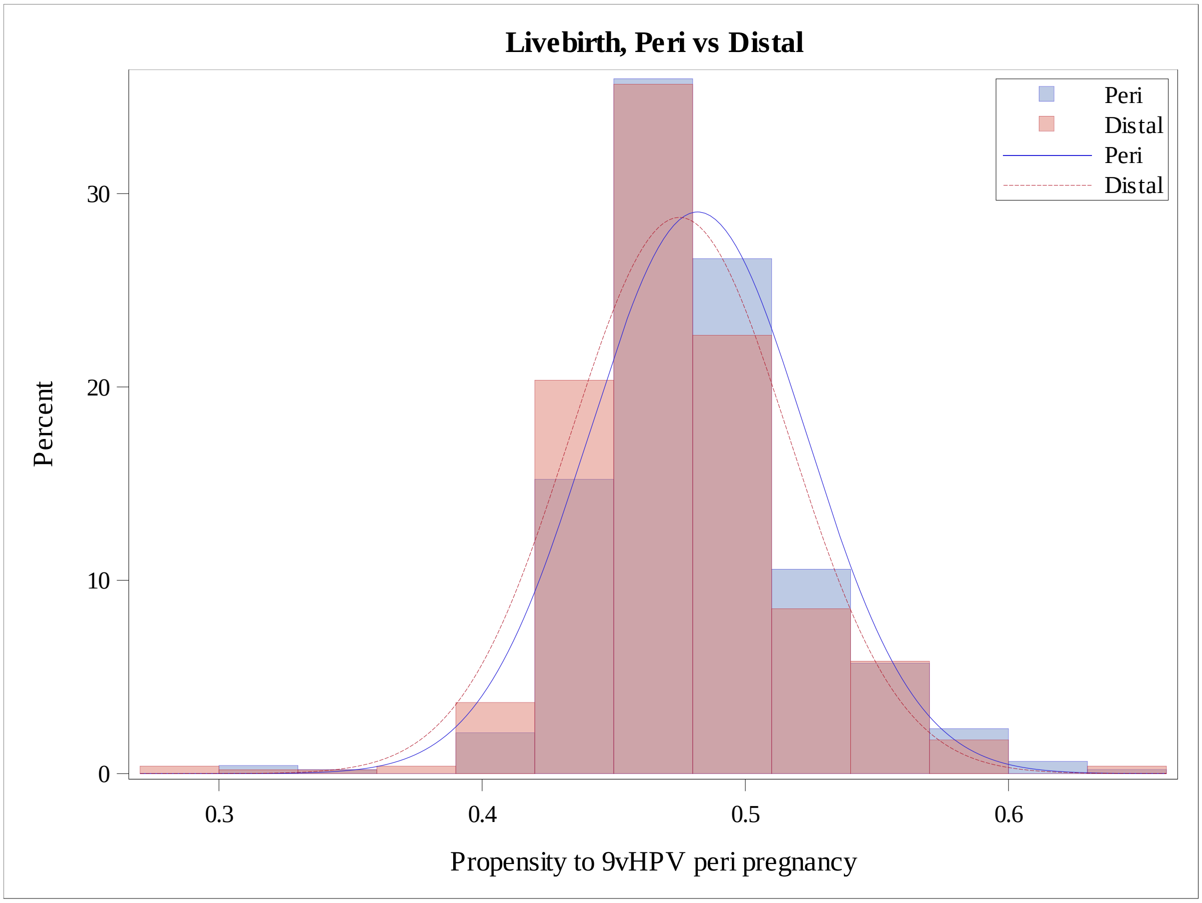

© 2021 Kharbanda EO et al. *JAMA Network Open*

eFigure 2. Standardized Differences Before and After IPW for Selected Risk Factors, Full Cohort

(a) During pregnancy vs Distal

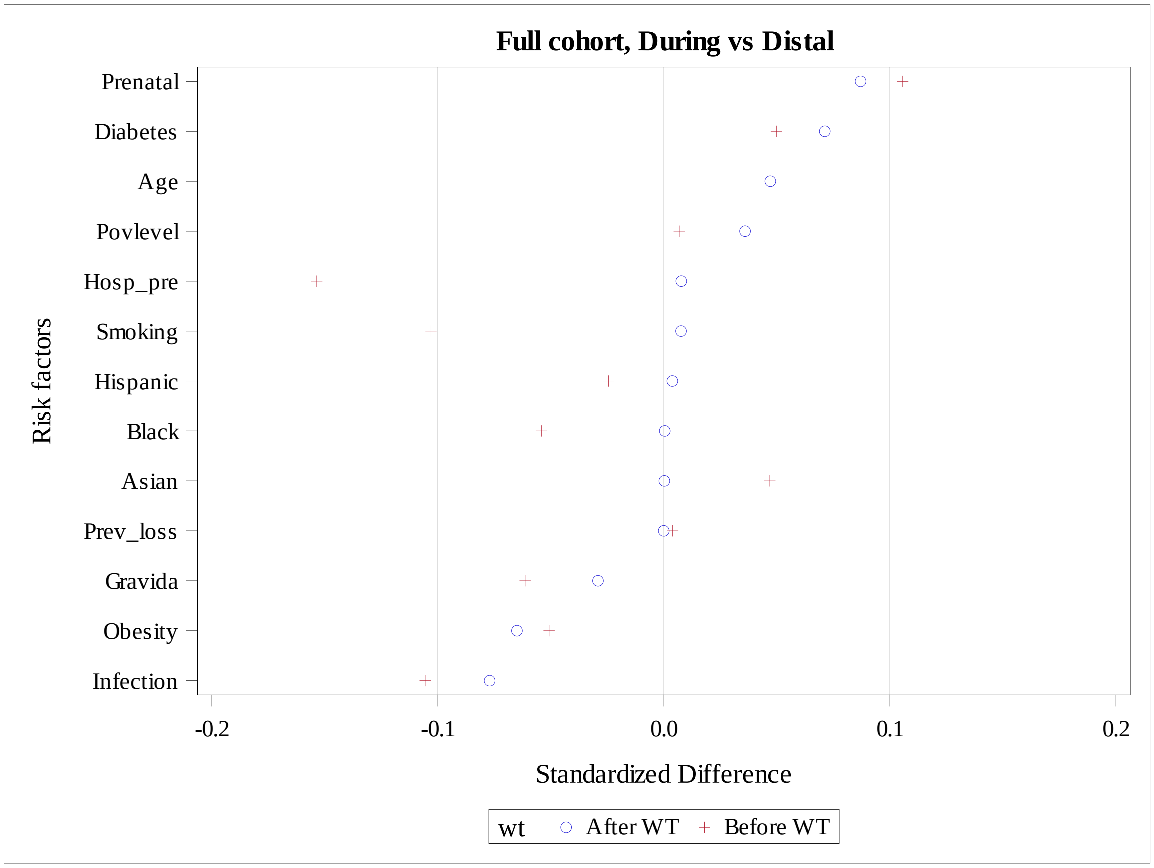

b) Peri-pregnancy vs Distal

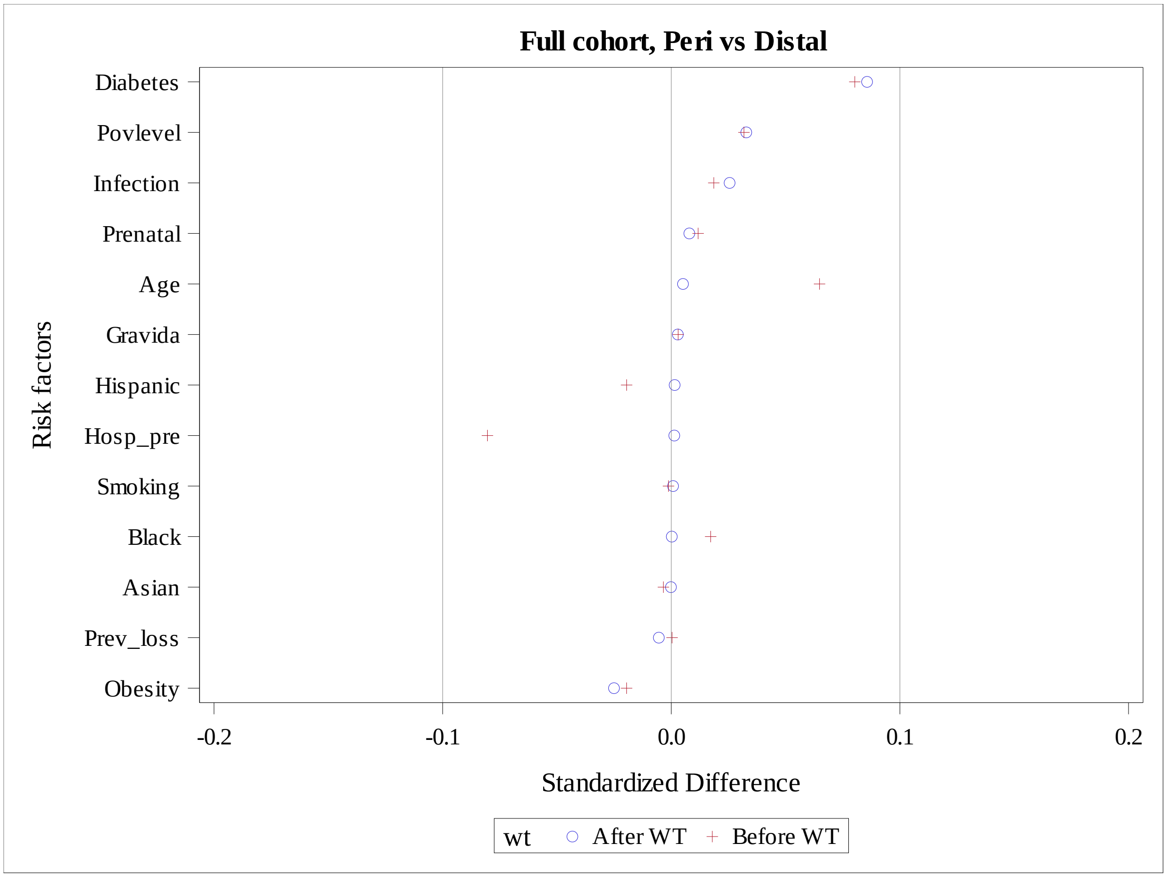

© 2021 Kharbanda EO et al. *JAMA Network Open*

c) Peri-pregnancy or During pregnancy vs Distal, for live birth cohort

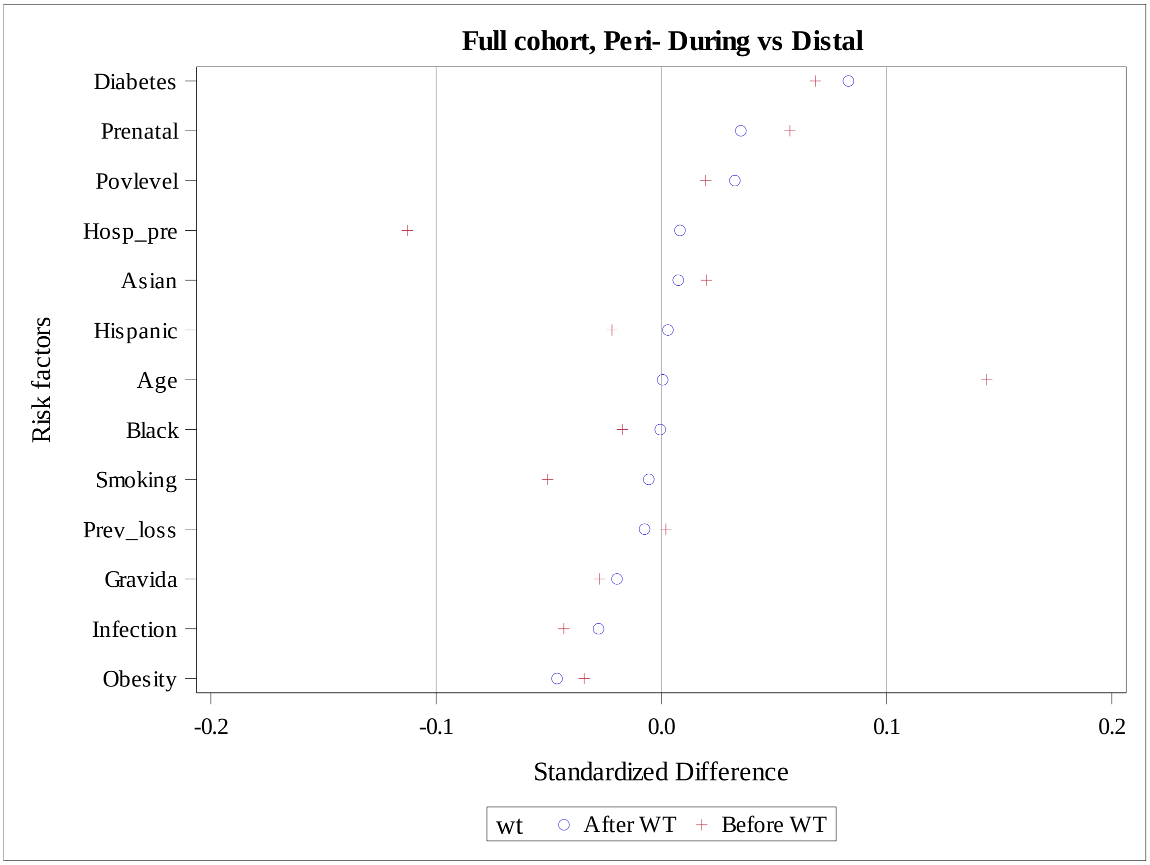

© 2021 Kharbanda EO et al. *JAMA Network Open*

d) During pregnancy vs Distal

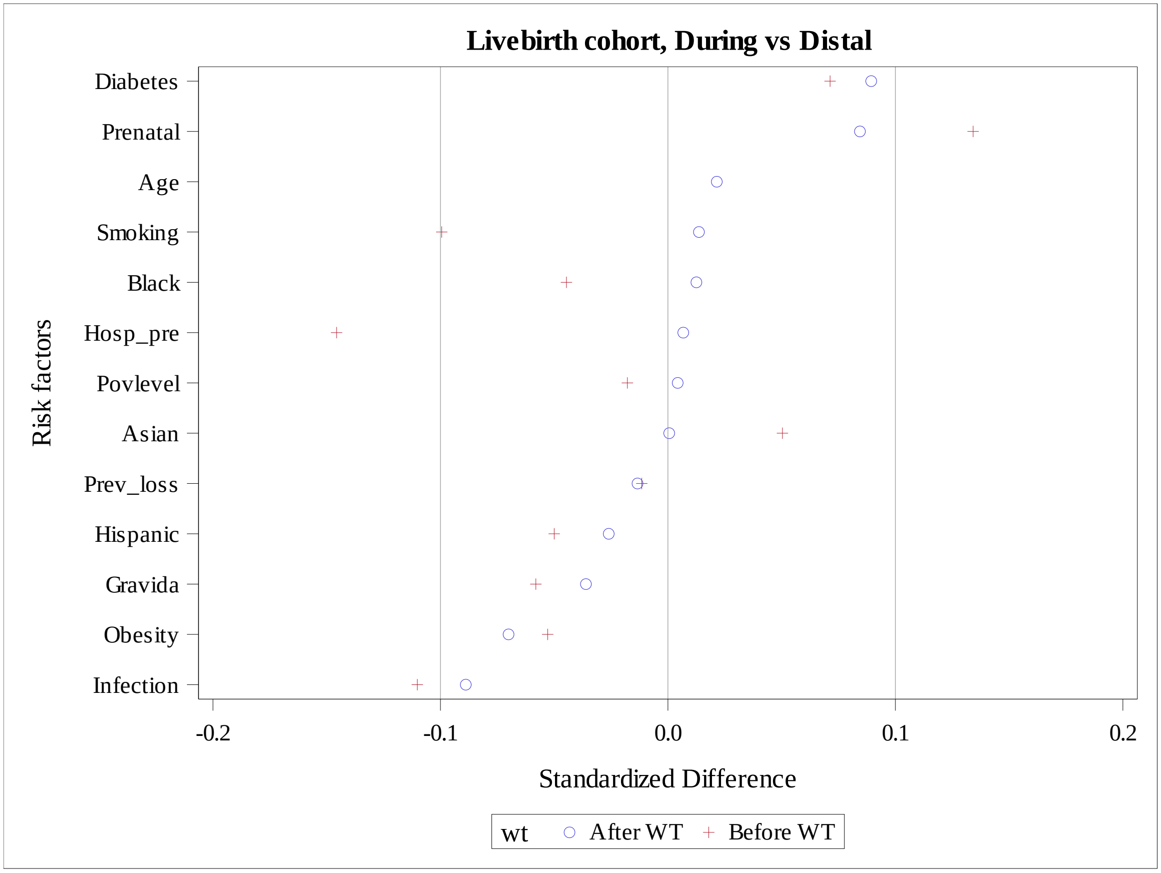

© 2021 Kharbanda EO et al. *JAMA Network Open*

e) Peri-pregnancy vs Distal

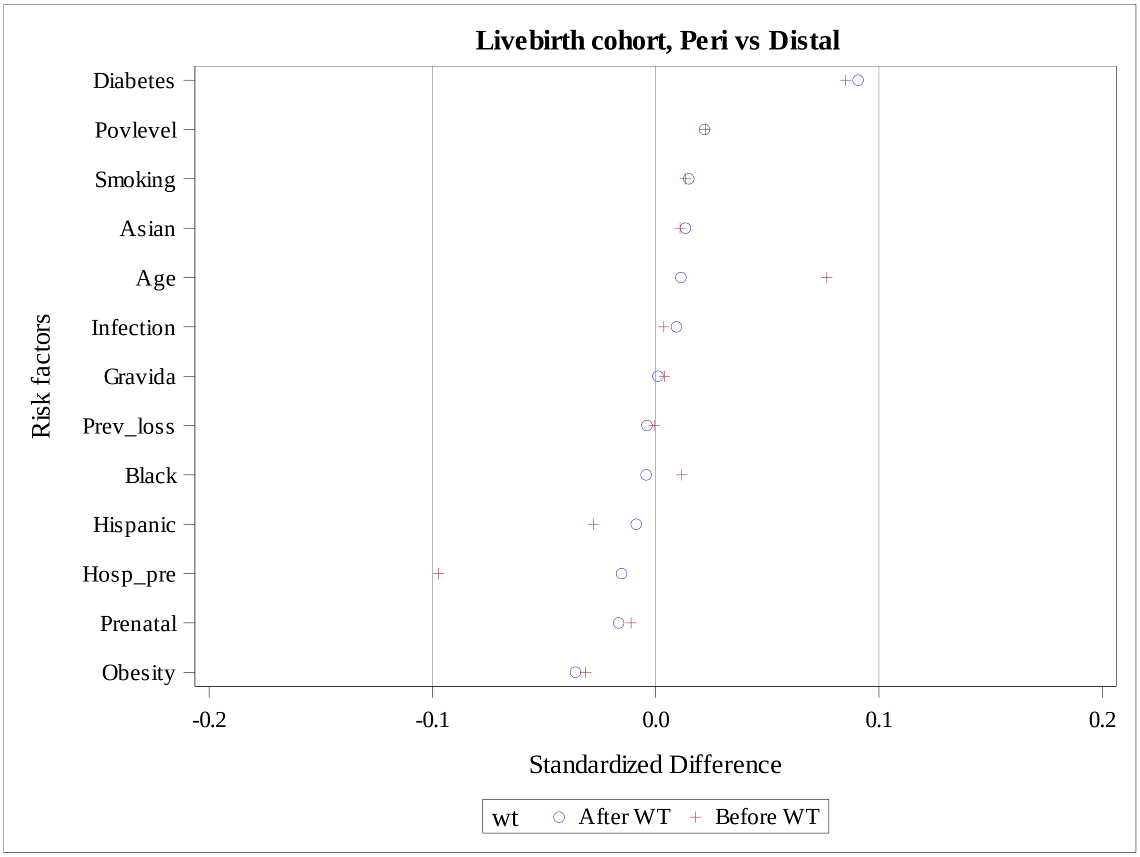

© 2021 Kharbanda EO et al. *JAMA Network Open*

f) Peri-pregnancy or During pregnancy vs Distal

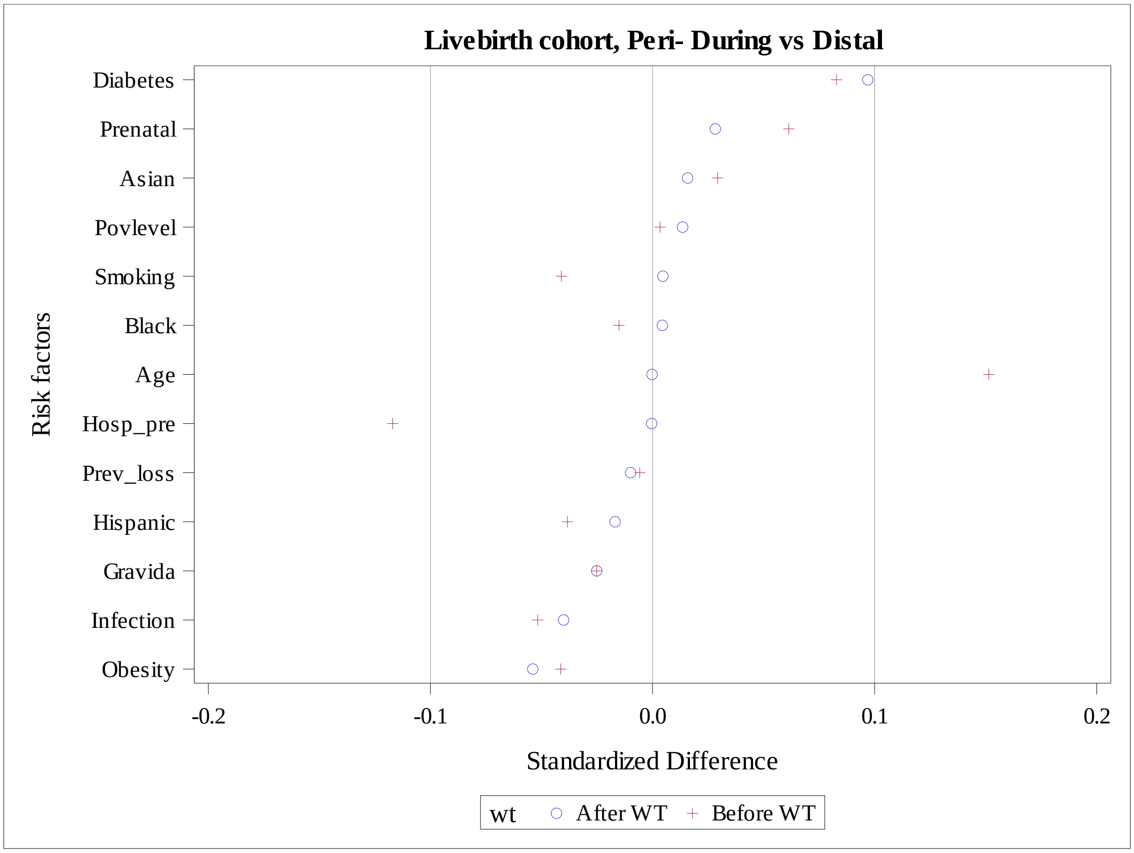

© 2021 Kharbanda EO et al. *JAMA Network Open*

eTable. List of Teratogenic and Abortifacient Medication Exclusions if Prescription Fill During Pregnancy or Within 8 Months of Last Menstrual Period

Drug Class	Drug Name	Drug Classification
Prostaglandin Analog	Mifepristone	Abortifacient
Prostaglandin Analog	Carboprost	Abortifacient
Prostaglandin Analog	Sulprostone	Abortifacient
Prostaglandin Analog	Gemeprost	Abortifacient
Prostaglandin Analog	Misoprostol	Abortifacient
Antineoplastic	Methotrexate	Abortifacient
Vitamin A Analog	Isotretinoin	Teratogenic
Vitamin A Analog	Bexarotene	Teratogenic
Vitamin A analog	Acitretin	Teratogenic
Immuosuppressant	Mycophenolate Mofetil	Teratogenic
Immunologic	Azathioprine	Teratogenic
Immunologic	Thalidomide	Teratogenic
Anticoagulant	Warfarin	Teratogenic
Mood Stabilizer	Lithium	Teratogenic
Antiarrhythmic	Amiodarone	Teratogenic
Antiarrhythmic	Dronedarone	Teratogenic
Anticonvulsant	Carbamazepine	Teratogenic
Anticonvulsant	Fosphenytoin	Teratogenic
Anticonvulsant	Mephobarbital	Teratogenic
Anticonvulsant	Phenobarbital	Teratogenic
Anticonvulsant	Phenytoin	Teratogenic
Anticonvulsant	Primidone	Teratogenic
Anticonvulsant	Topiramate	Teratogenic
Anticonvulsant	Valproic Acid and derivatives	Teratogenic

© 2021 Kharbanda EO et al. *JAMA Network Open*
